# Hemocompatibility and Long‐Term Outcomes in HeartWare Versus HeartMate II Versus HeartMate 3: Multicenter Real‐World Cohort

**DOI:** 10.1111/aor.70086

**Published:** 2026-01-04

**Authors:** Hamza H. H. Ben Nasir, Ahmed F. A. Mohammed, Omar Allham, Alish Kolashov, Yusuf Shieba, Lachmandath Tewarie, Bernd Panholzer, Ajay Moza, Assad Haneya, Rashad Zayat

**Affiliations:** ^1^ Department of Anesthesiology and Operative Intensive Care Klinikum Links der Weser gGmbH Bremen Germany; ^2^ Department of Cardiac Surgery RWTH University Hospital Aachen Germany; ^3^ Department of Cardiothoracic Surgery, Faculty of Medicine Qena University Qena Egypt; ^4^ Department of Cardiac Surgery, Heart Centre Dresden Technische Universität Dresden Dresden Germany; ^5^ Department of Cardiothoracic Surgery, Heart Centre Trier Barmherzigen Brueder Hospital Trier Germany; ^6^ Department of Cardiac Surgery University Hospital Schleswig‐Holstein Kiel Germany

**Keywords:** HeartMate 3, HeartMate II, HeartWare (HVAD), hemocompatibility‐related adverse events, left ventricular assist device

## Abstract

**Background:**

To compare overall and hemocompatibility‐related outcomes across third‐generation centrifugal‐flow HeartMate 3 (HM3) versus second‐generation axial‐flow HeartMate II (HMII) and centrifugal‐flow HeartWare (HVAD) in routine clinical practice.

**Methods:**

We conducted a multicenter observational cohort of adult LVAD recipients (*n* = 327: HVAD *n* = 112, HMII *n* = 142, HM3 *n* = 73). Baseline characteristics and perioperative variables were recorded. Overall survival was analyzed using Kaplan–Meier and Cox proportional hazards models with HM3 as reference. Hemocompatibility‐related adverse events (HRAE; pump thrombosis, ischemic stroke, major bleeding) were assessed with cumulative incidence functions (Aalen–Johansen) and Fine–Gray competing‐risk regression with death as a competing event; the hemocompatibility score (HCS) summarized event burden over follow‐up.

**Results:**

HM3 demonstrated superior long‐term survival compared with both HMII and HVAD in Kaplan–Meier analyses (log‐rank *p* < 0.001); this advantage persisted in multivariable Cox models. HRAE‐free survival was also higher with HM3 on competing‐risk analysis, driven by substantially lower pump thrombosis and fewer ischemic strokes relative to HMII and HVAD. Bleeding burden did not differ materially between devices, consistent with a class effect of continuous‐flow support. LDH‐based hemolysis markers and HCS distributions further favored HM3. Perioperative course and general postoperative complications were otherwise broadly comparable across devices.

**Conclusions:**

In this real‐world, multicenter cohort, HM3 was associated with better overall survival and a lower thromboembolic burden than HMII and HVAD, while bleeding risk remained similar. These findings support preferential selection of HM3 when device choice is feasible, and emphasize the need for targeted strategies to mitigate hemostatic complications across all LVADs.

## Graphical abstract

Multicenter real‐world cohort (*n* = 327) comparing LVADs: HeartMate 3 (HM3), HeartMate II (HMII), and HeartWare (HVAD). HM3 showed superior long‐term survival versus legacy devices, driven by fewer hemocompatibility‐related adverse events, especially pump thrombosis and ischemic stroke, while major bleeding was similar across devices. Findings support preferential HM3 selection in routine practice.

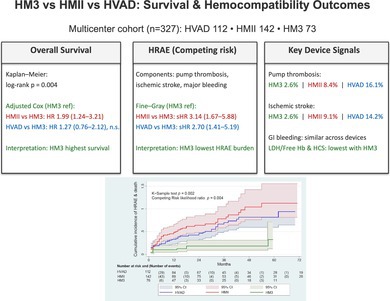

## 1 Introduction

Left ventricular assist devices (LVADs) serve as a crucial treatment option for patients suffering from advanced heart failure refractory to medical therapy [1, 2]. Despite significant advancements in device technology and patient care, hemocompatibility‐related adverse events (HRAEs) remain a major concern, contributing substantially to patient morbidity and mortality [3, 4]. These HRAEs, which include gastrointestinal bleeding, hemolysis, and thrombotic events such as pump thrombosis and stroke, frequently extend beyond the immediate postoperative period and are a leading cause of rehospitalization [5]. The increasing longevity of patients on LVAD support further underscores the critical importance of preventing and managing these complications [5].

The evolution of LVAD technology has led to the development of third‐generation devices, characterized by enhanced hemocompatibility [4, 6]. Among these, the fully magnetically levitated HeartMate 3 (HM3) has shown particularly promising results. Large clinical trials, such as the MOMENTUM 3, demonstrated that HM3 significantly reduced the incidence of HRAEs, including de novo pump thrombosis, ischemic and hemorrhagic strokes, and nonsurgical bleeding, when compared to the axial‐flow HeartMate II (HMII) LVAD [1]. Five‐year observational follow‐up data from the MOMENTUM 3 trial further confirmed the superiority of HM3 over HMII, showing better composite outcomes and higher overall survival, with significantly lower rates of device thrombosis, stroke, and bleeding [6]. Recent systematic reviews and meta‐analyses also indicate that HM3 is associated with the lowest risk of overall mortality, cerebrovascular accidents (CVA), other neurological events, pump thrombosis, and bleeding when compared to both HMII and HeartWare (HVAD) [7].

Despite the well‐documented advantages of HM3 over HMII, a comprehensive understanding of long‐term outcomes and HRAE profiles across all three most commonly implanted durable LVADs globally—HMII, HVAD, and HM3—remains incomplete [2, 8]. Direct, large‐scale randomized controlled trials comparing HM3 and HVAD have not yet been performed and are not anticipated [2, 9]. Existing comparative data are largely derived from single‐center retrospective studies or analyses of large registries, which often present challenges due to inconsistencies in patient selection, treatment protocols, follow‐up durations, and adverse event definitions, introducing potential biases [10]. Furthermore, while the HVAD has faced discontinuation due to technical issues and concerns about pump thrombosis and malfunctions, highlighting the importance of comparative data, its outcomes still warrant thorough evaluation alongside other devices in real‐world settings [8]. This critical gap in comparative long‐term, real‐world evidence across these three device types impedes optimal clinical decision‐making and patient risk stratification.

Therefore, this multicenter observational study aims to address this knowledge gap by providing a comprehensive comparison of HRAEs and long‐term outcomes among patients supported with the HeartWare (HVAD), HMII, and HM3 left ventricular assist devices in a real‐life patient cohort.

## 2 Methods

This research performed a retrospective analysis of patients who received LVAD implantation at three mid‐volume centers in Germany. Data from LVAD patients were anonymized and collected between 2014 and 2021. Demographic information, etiology of heart failure, device strategy, clinical characteristics, laboratory results, and adverse events were recorded at preimplantation and postimplantation intervals, specifically at discharge, 3 months, 6 months, and every 6 months thereafter for the duration of the device's life. The ethical oversight for this study was performed by the Institutional Review Board at RWTH University of Aachen (EK151/09). Adverse events and right heart failure were defined in accordance with the Mechanical Circulatory Support Academic Research Consortium [11]. Data collection ended in 2021 as this was the final year all three devices were in use and inter‐center cooperation could be maintained. Follow‐up was updated through September 2024. No patient was lost to follow‐up. Median follow‐up duration was 20.0 months (IQR 1.0–37.5), range 1–128 months.

### 2.1 Hemocompatibility‐Related Adverse Events (HRAEs)

We used the definition of HRAEs as suggested by Uriel et al. [12], these events included:

- Nonsurgical bleeding: Episodes of gastrointestinal or other nonsurgical bleeding occurring more than 30 days post‐implant.
- Neurological events: Incidents of stroke (either hemorrhagic or ischemic, whether disabling or nondisabling) or other neurological occurrences (e.g., transient ischemic attack, seizures) at any time.
- Thromboembolic events: Presence of suspected or confirmed pump thrombosis, as well as arterial thromboembolism, with or without organ involvement, at any time.

The Hemocompatibility Score (HCS) [12] was although calculated.

### 2.2 Protocol for Anticoagulation

At 12 h post‐implantation, when chest tube drainage decreased to 50 mL per hour and the coagulation profile normalized or approached normal levels, intravenous heparin infusion was initiated to maintain an activated partial thromboplastin time between 60 and 80 s. Aspirin was initiated at a dosage of 100 mg for the HM II and HM III groups, and 300 mg for the HVAD group, administered once daily following extubation. Following the removal of chest drains and the initiation of oral medication, phenprocoumon was administered to maintain an INR of 2.0–2.5 for HM II and HM III patients, and 2.5–3.0 for HVAD patients. The heparin infusion was maintained until the target INR range was achieved.

### 2.3 Statistical Analysis

Continuous variables are reported as median (IQR) and categorical variables as *n* (%). Baseline characteristics were compared across devices (HM3, HMII, HVAD) using Kruskal–Wallis (continuous) and Fisher's exact tests (categorical). To quantify baseline balance independently of sample size, we computed standardized mean differences (SMDs) versus HM3 for all variables (interpretation: < 0.10 negligible, 0.10–0.20 small, 0.20–0.50 moderate, ≥ 0.50 large). Overall survival was analyzed using Kaplan–Meier with log‐rank testing and Cox proportional hazards (HM3 reference), reporting hazard ratios (HRs) with 95% confidence intervals. The adjusted Cox model included female sex, INTERMACS level, albumin, hematocrit, SPAP, PCWP, bilirubin, and destination score. Proportional‐hazards assumptions were evaluated using Schoenfeld residuals.

HRAEs were analyzed in a competing‐risks framework with death as the competing event. We estimated cumulative incidence functions (Aalen–Johansen) and compared devices using Gray's test. Fine–Gray models (HM3 reference) with the same covariates reported subdistribution hazard ratios (sHRs) and 95% CIs. Analyses were complete case. Two‐sided *p* < 0.05 was considered statistically significant. Analyses used R (v4.x) with survival (Cox/KM), cmprsk (CIF/Fine–Gray), and ggplot2.

Adjusted effect estimates are tabulated in Table S1 and displayed as a forest plot in Figure S1; proportional‐hazards diagnostics are shown in Figure S2.

## 3 Results

### 3.1 Baseline Characteristics

Across 327 patients (HVAD *n* = 112, HMII *n* = 142, HM3 *n* = 73), demographic and risk profiles were broadly comparable (Table 1). Most variables showed negligible–small imbalance (SMD < 0.20). Differences were concentrated in etiology, several preoperative labs/hemodynamic, and operative exposure. Ischemic cardiomyopathy (ICM) was more frequent in HMII (73% vs. HVAD 50% and HM3 57%; Holm‐adjusted *p* ≈ 0.001; SMD 0.51), and nicotine use differed across cohorts (*p* < 0.001; SMD 0.79). Among continuous measures versus HM3, SMDs were moderate for albumin (0.41), hematocrit (0.36), SPAP (0.36), PCWP (0.33), bilirubin (0.32), urea (0.30), platelet count (0.30), destination score (0.25), and cardiac index (0.22); INR and INTERMACS 1 were borderline (≈0.20–0.21). Body size differed modestly (SMD 0.33). By contrast, female sex (SMD 0.13), pre‐op hemoglobin (0.17), LDH (0.13), EuroSCORE II (0.11), HMRS (0.16), and MRHFS (0.13) were well balanced. Operative metrics varied: cross‐clamp use/time and total operative time were longer mainly in HMII (pairwise Holm‐adjusted *p* < 0.001), and CPB time also differed (adjusted *p* ≤ 0.006 across key pairs). The frequency of concomitant procedures did not differ significantly across groups. CABG was performed in 21.4% of HVAD, 17.6% of HMII, and 20.5% of HM3 patients (*p* = 0.727). Aortic valve replacement occurred in 14.3%, 8.4%, and 10.9%, respectively (*p* = 0.337). Tricuspid valve interventions were more common in HVAD recipients (13.4% vs. HMII 5.6% and HM3 6.9%), although this difference did not reach statistical significance (*p* = 0.076). Implant approach also differed across devices. Minimally invasive left lateral thoracotomy was performed in 20 HVAD patients and 15 HM3 patients, whereas all remaining implants were carried out via median sternotomy.

**TABLE 1:** Baseline patient characteristics.

Variable	HVAD (*n* = 112)	HMII (*n* = 142)	HM3 (*n* = 73)	Test statistic	SMD vs. HM3
				*p*	
Total, *N* (%)	112 (34.3)	142 (43.4)	73 (22.3)		
Female	18 (16.1)	22 (15.5)	12 (16.4)	0.982	0.13
Age, years	67.0 (57.8, 72.2)	64.0 (57.0, 70.0)	63.0 (55.0, 70.0)	0.235	0.10
BSA, m^2^	2.0 (1.8, 2.2)	2.0 (1.8, 2.1)	2.1 (1.9, 2.2)	0.046	0.33
BMI, kg/m^2^	25.5 (23.1, 29.3)	26.3 (24.0, 29.0)	28.4 (25.6, 32.3)	0.001	0.51
ICM, *n* (%)	57 (50%)	105 (73%)	44 (57%)	0.001	0.51
BTT	50 (44.6)	48 (33.8)	29 (39.7)	0.209	0.12
DT	58 (51.8)	67 (47.2)	39 (53.4)	0.627	0.12
BTC	4 (3.6)	19 (13.4)	4 (5.5)	0.012	0.26
IDDM	11 (9.8)	22 (15.5)	14 (19.2)	0.183	0.27
PAD	16 (14.4)	33 (23.2)	13 (17.8)	0.198	0.13
Arterial hypertension	64 (57.1)	81 (57.0)	52 (71.2)	0.094	0.11
Nicotine	81 (72.3)	46 (32.4)	26 (35.6)	< 0.001	0.79
EF, %	20.0 (15.0, 23.5)	20.0 (15.0, 22.8)	20.0 (18.0, 25.0)	0.204	0.12
History of apoplex	12 (10.7)	8 (5.6)	10 (13.7)	0.120	0.29
Preop CKD					
0	65 (58.0)	86 (60.6)	39 (53.4)	0.137	0.17
I	0 (0.0)	7 (4.9)	2 (2.7)		0.13
II	17 (15.2)	15 (10.6)	7 (9.6)		0.17
III	23 (20.5)	20 (14.1)	19 (26.0)		0.15
IV	7 (6.2)	14 (9.9)	6 (8.2)		0.14
Preop dialysis	6 (5.4)	13 (9.2)	5 (6.8)	0.506	0.08
COPD GOLD					
0	98 (87.5)	106 (74.6)	53 (72.6)	< 0.001	0.39
I	12 (10.7)	4 (2.8)	8 (11.0)		0.36
II	2 (1.8)	24 (16.9)	7 (9.6)		0.37
III	0 (0.0)	7 (4.9)	2 (2.7)		0.27
IV	0 (0.0)	1 (0.7)	3 (4.1)		0.33
Pulmonary hypertension	53 (47.3)	78 (54.9)	48 (65.8)	0.048	0.37
Preop RHF	16 (14.3)	24 (16.9)	21 (28.8)	0.037	0.37
INTERMACS 1	14 (12.5)	24 (16.9)	7 (9.6)	0.301	0.21
INTERMACS 2	24 (21.4)	21 (14.8)	11 (15.1)	0.329	0.16
EuroSCORE II	13.8 (8.3, 28.0)	13.8 (7.5, 23.8)	13.8 (8.6, 23.8)	0.752	0.11
HMRS	1.8 (1.3, 2.5)	1.8 (1.3, 2.5)	1.9 (1.4, 2.5)	0.803	0.16
MRHFS	2.5 (0.0, 4.0)	2.5 (0.0, 4.0)	2.5 (0.0, 4.0)	0.682	0.13
Destination score	12.0 (8.0, 16.0)	12.0 (7.0, 16.0)	10.0 (6.0, 14.0)	0.267	0.25
Preoperative laboratory					
Hb, g/dL	10.8 (9.6, 12.0)	10.9 (9.8, 13.0)	11.4 (9.9, 12.5)	0.528	0.17
Hematocrit, %	32.5 (29.2, 36.0)	33.5 (29.3, 37.8)	35.0 (30.7, 38.7)	0.065	0.36
Platelet count, /nL	185.0 (136.8, 246.8)	188.5 (142.2, 245.2)	207.0 (172.0, 274.0)	0.047	0.30
Albumin, g/dL	3.2 (2.8, 3.6)	3.2 (2.6, 3.6)	3.4 (2.9, 4.0)	0.067	0.41
INR	1.3 (1.2, 1.4)	1.2 (1.1, 1.4)	1.2 (1.1, 1.4)	0.053	0.20
AST, U/L	28.0 (21.3, 41.2)	33.0 (23.0, 48.8)	30.0 (21.0, 49.0)	0.192	0.06
ALT, U/L	24.7 (17.0, 46.0)	29.0 (21.0, 52.0)	26.0 (17.0, 69.0)	0.163	0.15
LDH, U/L	241.0 (195.8, 325.2)	229.5 (195.8, 336.8)	234.0 (212.0, 327.0)	0.606	0.13
Urea, mg/dL	55.0 (32.0, 92.5)	56.0 (38.5, 66.0)	54.0 (38.0, 66.0)	0.504	0.30
Bilirubin, mg/dL	0.7 (0.5, 1.4)	0.7 (0.5, 1.2)	0.6 (0.4, 1.1)	0.122	0.32
Cardiac index, L/min/m^2^	1.9 (1.6, 2.3)	1.9 (1.7, 2.3)	1.8 (1.5, 2.1)	0.304	0.22
CVP, mmHg	14.0 (10.0, 18.0)	14.0 (10.0, 17.0)	14.0 (11.0, 18.0)	0.543	0.14
Mean PAP, mmHg	31.7 (24.8, 38.9)	31.6 (25.7, 36.9)	31.0 (24.0, 38.0)	0.842	0.06
SPAP, mmHg	49.2 (39.0, 58.1)	46.0 (38.7, 57.4)	43.0 (34.0, 54.0)	0.070	0.36
PCWP, mmHg	20.0 (16.0, 25.2)	19.0 (12.0, 25.0)	19.0 (12.0, 24.0)	0.083	0.33
OP time, min	234.0 (187.8, 286.2)	291.0 (242.0, 362.5)	219.0 (182.0, 305.0)	< 0.001	0.69
CPB time, min	111.0 (95.0, 132.0)	131.0 (101.0, 178.8)	100.5 (78.0, 146.5)	< 0.001	0.52
Cross clamp, min	64.5 (0.0, 77.5)	0.0 (0.0, 0.0)	0.0 (0.0, 0.0)	< 0.001	1.17
CABG	24 (21.4)	25 (17.6)	15 (20.5)	0.727	0.022
AVR	16 (14.3)	12 (8.4)	8 (10.9)	0.337	0.100
TVR	15 (13.4)	8 (5.6)	5 (6.9)	0.076	0.217

*Note:* Data are presented as *n* (%) or median (interquartile range).

Abbreviations: ALT, alanine aminotransferase; AST, aspartate aminotransferase; AVR, aortic valve replacement; BMI, body mass index; BSA, body surface area; BTC, bridge to candidacy; BTT, bridge to transplant; CABG, coronary aortic bypass graft; COPD, chronic obstructive pulmonary disease; CPB, cardiopulmonary bypass; CVP, central venous pressure; DT, destination therapy; EF, ejection fraction; EuroSCORE II, European System for Cardiac Operative Risk Evaluation II; HM3, HeartMate 3; HMII, HeartMate II; HMRS, HeartMate II Risk Score; HVAD, HeartWare Ventricular Assist Device; ICM, ischemic cardiomyopathy; IDDM, insulin‐dependent diabetes mellitus; INR, international normalized ratio; INTERMACS, Interagency Registry for Mechanically Assisted Circulatory Support; LDH, lactate dehydrogenase; mPAP, mean pulmonary artery pressure; MRHFS, Michigan Right Heart Failure Score; OP time, operative time; PAD, peripheral arterial disease; PCWP, pulmonary capillary wedge pressure; RHF, right heart failure; SPAP, systolic pulmonary artery pressure.

### 3.2 Postoperative Outcomes

The duration of inotropic support differed significantly, with HM3 patients requiring the shortest median duration (*p* = 0.010). Notably, the incidence of right ventricular assist device (RVAD) implantation was higher in the HeartMate II group (*p* = 0.041). The occurrence of HRAEs (a composite of nonsurgical bleeding, neurological events, and thromboembolic events) differed significantly among the device groups (*p* = 0.037). Pump thrombosis and ischemic stroke were significantly more frequent in HVAD patients compared to HMII and HM3 recipients (*p* = 0.0004 and *p* = 0.027, respectively). Rates of gastrointestinal bleeding and hemorrhagic stroke did not differ significantly across groups (*p* = 0.708 and *p* = 0.269, respectively). Overall, the HVAD group had the highest proportion of patients experiencing at least one HRAE (50%) compared to HMII (45.8%) and HM3 (31.5%). Free hemoglobin and LDH levels also showed significant differences across groups, indicating variable hemolysis profiles (*p* = 0.0011 and *p* = 0.0021).

The hemocompatibility score (HCS) distribution differed markedly: the HMII group showed higher rates of cumulative HCS ≥ 3 (*p* < 0.0012) and ≥ 5 (*p* = 0.0412), reflecting more severe adverse hemocompatibility events compared to HVAD and HM3. Other complications, including delirium, pneumonia, septic shock, and bleeding events, did not differ significantly across groups (*p* > 0.05).

Overall, these findings suggest meaningful differences in pump thrombosis, stroke, hemolysis markers, and hemocompatibility burden among the devices, while most general postoperative complications remained comparable (Table 2).

**TABLE 2:** Postoperative outcomes and hemocompatibility‐related adverse events.

Variable	HVAD (*n* = 112)	HMII (*n* = 142)	HM3 (*n* = 73)	Test statistic
				*p*
ICU stay (days)	7 (4, 13)	6 (4, 13)	6 (3, 10.5)	0.220
iNO duration (h)	0 8 (0, 24)	6 (0, 24)	7 (0, 48)	0.139
Inotropes (h)	87.5 (34, 232)	57.5 (23, 238)	35 (16, 147)	0.010
Delirium, *n* (%)	21 (18.7%)	27 (11.9%)	14 (18.4%)	0.260
Pneumonia, *n* (%)	35 (31.2%)	46 (32.4%)	25 (32.8%)	0.968
Septic shock, *n* (%)	28 (25%)	45 (31.9%)	13 (17.5%)	0.071
RHF, *n* (%)	15 (13.4%)	24 (16.9%)	8 (10%)	0.417
RVAD, *n* (%)	4 (3.6%)	14 (9.8%)	2 (2.6%)	0.041
GIB, *n* (%)	33 (29.4%)	45 (31.7%)	20 (26.3%)	0.708
Pump thrombosis, *n* (%)	18 (16.1%)	12 (8.4%)	2 (2.6%)	0.008
Ischemic stroke, *n* (%)	16 (14.2%)	13 (9.1%)	2 (2.6%)	0.027
Hemorrhagic stroke, *n* (%)	8 (7.2%)	4 (2.8%)	4 (5.2%)	0.269
HRAE	56 (50%)	65 (45.8%)	24 (31.5%)	0.037
Free Hb, mg/dL	53 (38.8, 69)	40.5 (24, 59)	42 (27.3, 66.6)	0.001
LDH, U/L	348 (276, 871)	390 (317, 572)	308 (248, 428)	0.002
ALT, U/L	28.6 (15.2, 46.5)	30.0 (20.9, 50.0)	29.2 (19.7, 45.8)	0.424
HCS	0 (0, 10)	0 (0, 9)	0 (0, 9)	0.011
HCS = 1	21 (18.7%)	4 (2.8%)	7 (9.2%)	< 0.001
HCS = 2	9 (8.0%)	1 (0.7%)	1 (1.3%)	0.003
HCS = 3	9 (8.0%)	36 (25.3%)	3 (3.9%)	< 0.001
HCS = 4	7 (6.2%)	8 (5.6%)	4 (5.2%)	0.957
HCS ≥ 5	4 (3.5%)	14 (9.8%)	2 (2.6%)	0.041

*Note:* Data are presented as *n* (%) or median (interquartile range).

Abbreviations: ALT, alanine aminotransferase; GIB, gastrointestinal bleeding; Hb, hemoglobin; HCS, hemocompatibility score; HRAE, hemocompatibility‐related adverse events; ICU, intensive care unit; iNO, inhaled nitric oxide; LDH, lactate dehydrogenase; RHF, right heart failure; RVAD, right ventricular assist device.

### 3.3 Overall Survival (Cox Models)

**FIGURE 1:**
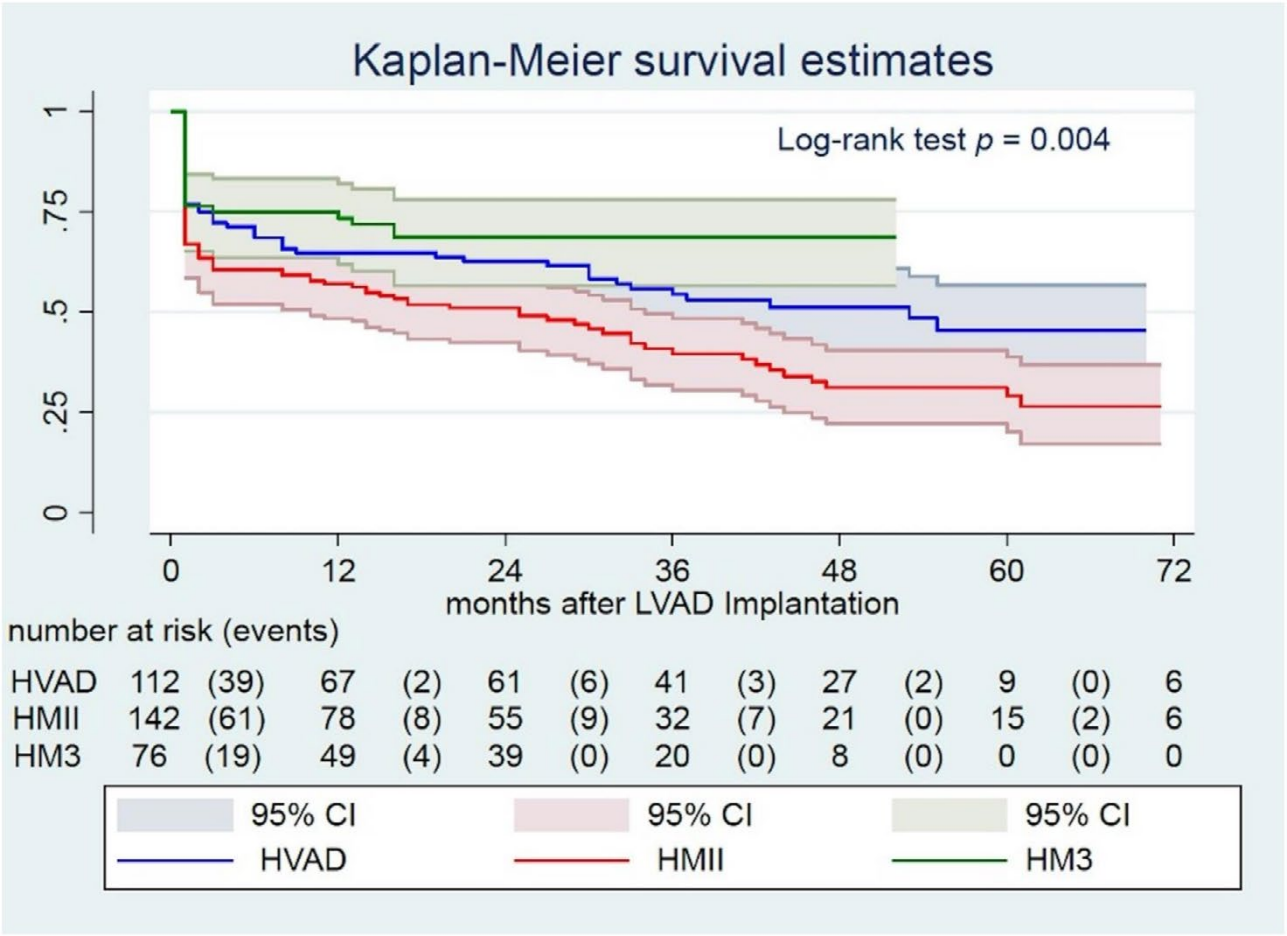
Kaplan–Meier survival by device. HM3, HeartMate 3; HMII, HeartMate II; HVAD, HeartWare. [Color figure can be viewed at wileyonlinelibrary.com]

Kaplan–Meier survival analysis demonstrated significant differences in overall survival among the device groups (*p* = 0.004). HM3 recipients had the highest survival probability throughout follow‐up, whereas HVAD patients had the lowest, with HMII showing intermediate survival, reflecting improved long‐term outcomes with the third‐generation LVAD (Figure 1). During follow‐up, 57 patients underwent heart transplantation and 7 patients were explanted following myocardial recovery; these patients were censored accordingly.

Using HM3 as the reference, unadjusted Cox models showed higher mortality with HMII (HR 2.15, 95% CI 1.35–3.44; *p* = 0.0013) and no significant difference for HVAD (HR 1.37, 0.83–2.26; *p* = 0.218). In the adjusted model (covariates: female sex, INTERMACS level, pre‐op albumin, hematocrit, SPAP, PCWP, bilirubin, destination score), the association for HMII persisted (HR 1.99, 1.24–3.21; *p* = 0.0045), while HVAD remained nonsignificant (HR 1.27, 0.76–2.12; *p* = 0.353). Full adjusted estimates are provided in Table S1 and visualized in Figure S1. The formal Schoenfeld residual test suggested a potential violation of the proportional hazard's assumption for the device variable (*p* = 0.041). However, visual inspection of the residuals plot (Figure S2) showed no clinically meaningful, systematic deviation from proportionality over time. Therefore, the Cox model was retained as an appropriate representation of the overall hazard.

### 3.4 Hemocompatibility‐Related Adverse Events (Competing Risk)

**FIGURE 2:**
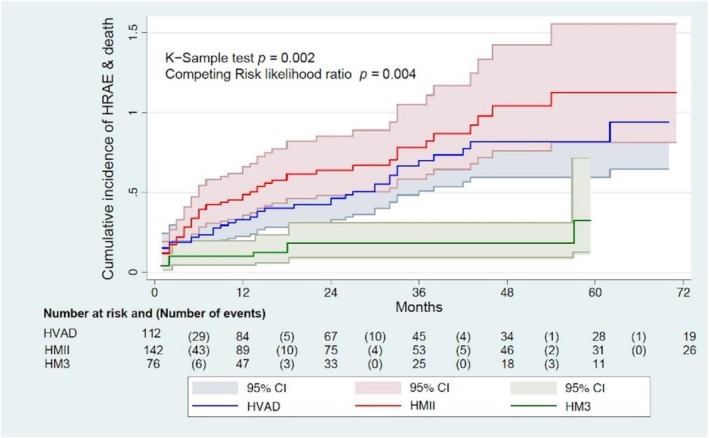
Cumulative incidence of HRAEs (Aalen–Johansen) + Gray's test. HM3, HeartMate 3; HMII, HeartMate II; HRAEs, hemocompatibility‐related adverse events; HVAD, HeartWare. [Color figure can be viewed at wileyonlinelibrary.com]

With death as the competing event, Fine–Gray models (HM3 reference) showed higher subdistribution hazards for the composite HRAE with both legacy devices. Unadjusted sHRs were 3.07 for HMII (95% CI 1.63–5.78; *p* < 0.001) and 2.89 for HVAD (1.52–5.49; *p* < 0.01). After adjustment using the same covariate set as the Cox model, the associations remained significant: HMII sHR 3.14 (1.67–5.88; *p* < 0.001) and HVAD sHR 2.70 (1.41–5.19; *p* = 0.00284); full adjusted subdistribution results are reported in Table S1 and Figure S1. These adjusted competing‐risk results align with the cumulative incidence curves (Aalen–Johansen) and Gray's test reported Figure 2.

## 4 Discussion

This multicenter, real‐world cohort study provides a comprehensive comparison of long‐term outcomes and HRAEs among patients supported by three distinct generations of continuous‐flow LVADs: the HVAD, HMII, and HM3. Our analysis demonstrates a clear hierarchy of device performance, with the latest‐generation centrifugal‐flow HM3 showing superior outcomes in survival and freedom from HRAEs compared to both the axial‐flow HMII and the centrifugal‐flow HVAD.

The principal findings of this study emphasize the clinical impact of technological evolution in LVAD design. Patients receiving the HM3 experienced significantly better overall survival and HRAE‐free survival compared to those with an HVAD or HMII. This was achieved by a markedly lower incidence of severe HRAEs. Specifically, the rate of pump thrombosis was exceptionally low in the HM3 group (2.6%) compared to the HMII (8.4%) and particularly the HVAD (16.1%). Similarly, ischemic stroke was least frequent in HM3 recipients (2.6%) versus HMII (9.1%) and HVAD (14.2%). The lowest cumulative Hemocompatibility Score (HCS) reflects the superior composite outcome for the HM3, which is a result of these individual event rates, and indicates a much lower overall burden of severe adverse events. Conversely, the HVAD cohort demonstrated the highest rates of pump thrombosis and ischemic stroke, contributing to its poorer overall performance.

Consistent with our results, the study by Pagani et al. [13] of a large US administrative dataset demonstrated that the HeartMate 3 was associated with enhanced survival, decreased healthcare utilization, and lower costs than other commercially available LVADs [13].

Conversely, our results contrast with findings from Zhigalov et al. [14], who reported no difference in short‐term survival. However, their study was limited by a small cohort (*n* = 108, 17 HM3) and did not address HRAE‐free survival.

### 4.1 The Device‐Level Pattern We Observed Is Biologically Plausible

HMII's axial‐flow design with mechanical bearings introduces additional blood–bearing contact and sustained shear, conditions that promote acquired von Willebrand factor (vWF) abnormalities, platelet dysfunction, and thrombosis/embolism risk [15]. HVAD's centrifugal architecture reduces bearing contact but still generates high shear and, in some eras, performance issues that may have amplified thrombotic susceptibility [16, 17]. In contrast, HM3's fully magnetically levitated rotor, broader blood path, and artificial pulse are intended to lower stasis and peak shear, aligning with the lower thromboembolic burden we observed [18]. The longer cross‐clamp times observed in HVAD recipients likely reflect the higher frequency of concomitant intracardiac procedures and the more frequent use of minimally invasive thoracotomy approaches in this cohort, both of which can necessitate or prolong aortic clamping.

Our findings on LDH levels, a biomarker for hemolysis, further validate these engineering principles, with the lowest levels seen in the low‐shear HM3 and the highest in the high‐shear HMII [19].

Severe bleeding occurs in 11%–30% of patients with axial‐flow LVADs [20, 21] and is also prevalent among those with centrifugal‐flow LVADs [22], which significantly decrease thrombotic complications linked to axial‐flow devices [23]. The nonphysiological continuous blood flow of axial‐flow LVADs is suspected to contribute to the increased incidence of gastrointestinal bleeding. Proposed mechanisms include acquired von Willebrand factor deficiency and the formation of angiodysplasia [24]. The issue was resolved in the development of the HM3, with a Fully Magnetically Levitated design and the feature which produces an artificial pulse by periodically altering the pump's speed. This results in the ongoing opening and closing of the aortic valve, thereby preventing blood stagnation in this region [25].

Interestingly, while thrombotic complications differed significantly in our study, the rates of gastrointestinal bleeding were comparable across all three devices. This could be in part explained by the fact that angiopoietin‐1 (Ang1) and angiopoietin‐2 (Ang2) are increased immediately post HMII as well as HM3 implantation, as shown by Woelke et al. [26], who found a correlation between the Ang1 and Ang2 with increased bleeding incidence. Kim et al. [27] also found that patients with elevations in serum Angiopoietin‐2 and TNF‐α at baseline before LVAD implantation demonstrated increased bleeding events after LVAD implantation. Another possible explanation suggests that bleeding may be a class effect of continuous‐flow support, driven more by the required systemic anticoagulation and the physiological effects of nonpulsatile flow, such as acquired von Willebrand Syndrome, rather than by specific pump mechanics [4].

The findings from this research align substantially with and support the outcomes of the MOMENTUM 3 trial, which is the largest randomized controlled trial of LVADs conducted to date. The 5‐year follow‐up data from MOMENTUM 3 demonstrated the superiority of the HM3 compared to the HMII, indicating significantly higher overall survival rates and reduced incidence of debilitating stroke or reoperation, attributed to a significant decrease in pump thrombosis, stroke, and bleeding events [6].

The HCS data from our study, showing a lower burden of severe events with the HM3, also aligns with a key secondary analysis from MOMENTUM 3, which reported greater freedom from HRAEs and a trend toward a lower net HCS for the HM3 compared to the HMII [12]. Furthermore, systematic reviews and meta‐analyses comparing the three devices have similarly concluded that the HM3 is associated with the lowest risk of mortality, pump thrombosis, and cerebrovascular events [7]. The high rates of pump thrombosis and neurological events we observed with the HVAD are consistent with the safety concerns and post‐market surveillance data that led to its eventual market withdrawal in 2021.

The results of this study have significant clinical implications for device selection and patient management. The proven effectiveness of the HM3 in lowering serious blood clot issues confirms it as the best treatment option for long‐term LVAD therapy. With the withdrawal of the HVAD and the clear advantages over the HMII, the HM3 is currently the only durable LVAD with FDA approval and widespread use, a status strongly supported by our data. These findings are crucial for informing discussions with patients and families regarding the expected risks and benefits of LVAD therapy, highlighting that while risks remain, the likelihood of severe device‐related complications has been substantially reduced with modern technology. The persistent challenge of gastrointestinal bleeding across all devices, however, emphasizes the continued importance of meticulous blood pressure management and careful anticoagulation monitoring in all LVAD recipients.

### 4.2 Limitations

Limitations include the retrospective, multicenter design; complete‐case analyses; and potential residual confounding despite prespecified adjustment. HRAEs were modeled with death as the competing event; transplant/explant were censored. Event adjudication was not centralized, and time‐updated exposures (e.g., anticoagulation intensity) were unavailable. Event‐specific anticoagulation data (INR at time of event, duration of anticoagulation interruption, or heparin holds) were not uniformly available across centers, preventing assessment of whether thromboembolic events occurred during subtherapeutic anticoagulation. HM3 sample size was smaller than legacy devices, which may reduce precision for some contrasts.

### 4.3 Future Directions

While our study confirms the superior hemocompatibility of the HM3, it also highlights areas requiring further investigation. The persistent issue of gastrointestinal bleeding across all generations indicates a “hemocompatibility ceiling” for existing technologies. Future research should focus on novel strategies to mitigate bleeding risk, such as the development of more targeted antithrombotic therapies, including direct oral anticoagulants, which are currently under investigation [28]. Further studies are also needed to optimize management strategies for other complications that remain prevalent, such as right heart failure and driveline infections. Finally, long‐term, prospective registries will be essential to continue monitoring the performance of the HM3 and to identify any late‐emerging adverse events, ensuring the continued advancement of care for patients with end‐stage heart failure.

## 5 Conclusion

In this multicenter real‐world cohort, the HM3 demonstrated clear superiority in safety and efficacy over the HMII and HVAD devices. The HM3 was associated with the lowest incidence of pump thrombosis and ischemic stroke, resulting in a significantly better overall hemocompatibility profile, as reflected by the lowest cumulative Hemocompatibility Score (HCS). Notably, the prevalence of bleeding events did not differ significantly among the three cohorts, suggesting this remains a class effect of continuous‐flow support. This superior safety profile translated directly into the highest long‐term HRAE‐free survival for HM3 patients. These findings confirm the clinical benefits of third‐generation technology and reinforce the HM3 as the current standard of care for durable LVAD therapy, offering improved safety and survival for patients with advanced heart failure.

## Author Contributions

**Hamza H. H. Ben Nasir:** writing – original draft, data curation, formal analysis, data interpretation, investigation, statistics, visualization, draft the manuscript. **Ahmed F. A. Mohammed:** conceptualization methodology, data curation, visualization, formal analysis, investigation, writing – review and editing. **Omar Allham:** resources, data curation, writing – review and editing. **Alish Kolashov:** visualization, resources, writing – review and editing. **Yusuf Shieba:** investigation, writing – review and editing. **Lachmandath Tewarie:** project administration, writing – review and editing. **Bernd Panholzer:** data curation, writing – review and editing. **Ajay Moza:** supervision, methodology, writing – review and editing. **Assad Haneya:** conceptualization, supervision, validation, writing – review and editing. **Rashad Zayat:** conceptualization, project administration, supervision, validation, writing – review and editing.

## Funding

The authors have nothing to report.

## Conflicts of Interest

The authors declare no conflicts of interest.

## Supporting information


**Figure S1:** aor70086‐sup‐0001‐FigureS1.jpg.

**Figure S2:** aor70086‐sup‐0002‐FigureS2.jpg.

**Table S1:** aor70086‐sup‐0003‐TableS1.docx.

## Data Availability

The data that support the findings of this study are available on request from the corresponding author. The data are not publicly available due to privacy or ethical restrictions.
