# Supplementary figures and images for: Hemocompatibility and Long‐Term Outcomes in HeartWare Versus HeartMate II Versus HeartMate 3: Multicenter Real‐World Cohort
Source: Artif Organs. 2026 Jan 4;50(4):580–9. doi: 10.1111/aor.70086 (PMC13125387; doi:10.1111/aor.70086)

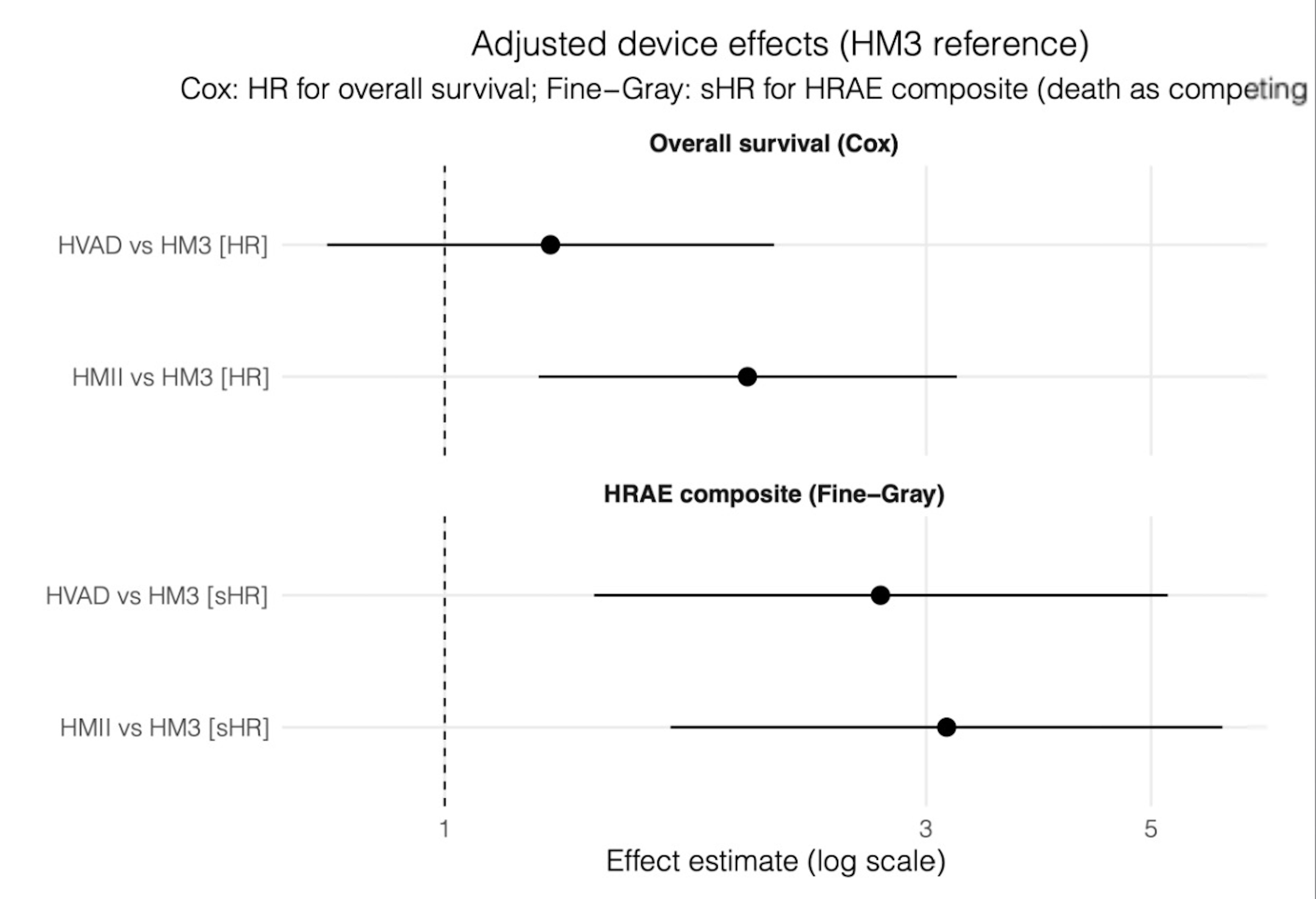

Supplement: Supplementary file 1 — Figure S1: aor70086‐sup‐0001‐FigureS1.jpg. [file AOR-50-580-s001.jpg]

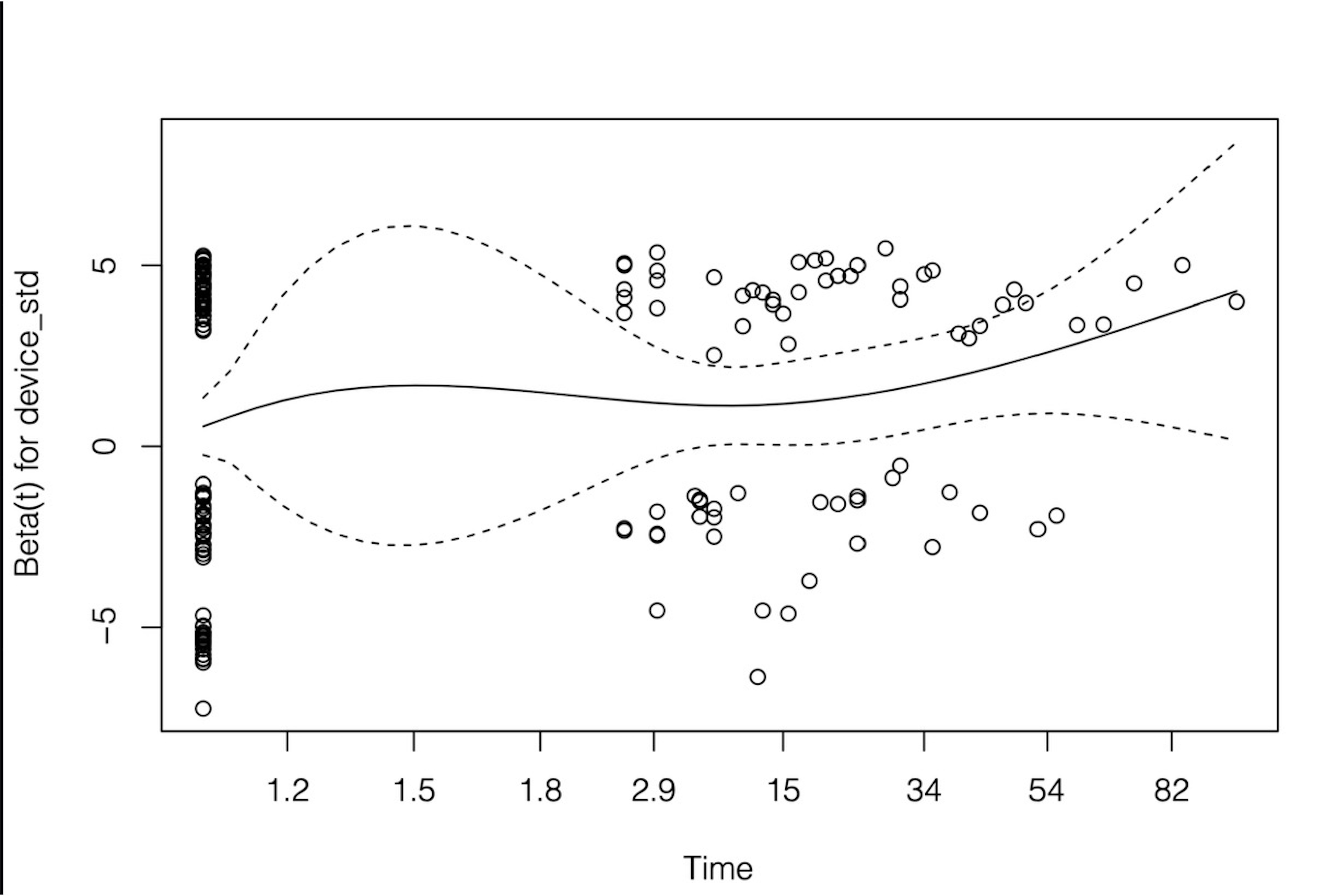

Supplement: Supplementary file 2 — Figure S2: aor70086‐sup‐0002‐FigureS2.jpg. [file AOR-50-580-s003.jpg]
