# Supplementary material for: Hemocompatibility and Long‐Term Outcomes in HeartWare Versus HeartMate II Versus HeartMate 3: Multicenter Real‐World Cohort
Source: Artif Organs. 2026 Jan 4;50(4):580–9. doi: 10.1111/aor.70086 (PMC13125387; doi:10.1111/aor.70086)
Supplement: Supplementary file 3 — Table S1: aor70086‐sup‐0003‐TableS1.docx. [file AOR-50-580-s002.docx]

# Supplementary Table S1. Adjusted device effects (Cox HR and Fine–Gray sHR)

| **Model / Outcome** | **Contrast (ref = HM3)** | **Effect** | **Estimate** | **95% CI** | **p-value** |
| --- | --- | --- | --- | --- | --- |
| Overall survival (Cox) | HMII vs HM3 | HR | 1.99 | 1.24–3.21 | 0.004 |
| Overall survival (Cox) | HVAD vs HM3 | HR | 1.27 | 0.76–2.12 | 0.353 |
| HRAE composite (Fine–Gray) | HMII vs HM3 | sHR | 3.14 | 1.67–5.88 | <0.001 |
| HRAE composite (Fine–Gray) | HVAD vs HM3 | sHR | 2.70 | 1.41–5.19 | 0.003 |

**Notes:** Models adjusted for: female_bin, intermacs_level, pre_albumin, pre_hematocrit, spap, pcwp, pre_bilirubin, destination_score. Fine–Gray uses death as the competing event. HM3 is the reference group. HR = hazard ratio; sHR = subdistribution hazard ratio.
